# Using meta-predictions to identify experts in the crowd when past performance is unknown

**DOI:** 10.1371/journal.pone.0232058

**Published:** 2020-04-24

**Authors:** Marcellin Martinie, Tom Wilkening, Piers D. L. Howe

**Affiliations:** 1 Melbourne School of Psychological Sciences, The University of Melbourne, Parkville, Victoria, Australia; 2 Department of Economics, The University of Melbourne, Parkville, Victoria, Australia; Ulm University, GERMANY

## Abstract

A common approach to improving probabilistic forecasts is to identify and leverage the forecasts from experts in the crowd based on forecasters’ performance on prior questions with known outcomes. However, such information is often unavailable to decision-makers on many forecasting problems, and thus it can be difficult to identify and leverage expertise. In the current paper, we propose a novel algorithm for aggregating probabilistic forecasts using forecasters’ meta-predictions about what other forecasters will predict. We test the performance of an extremised version of our algorithm against current forecasting approaches in the literature and show that our algorithm significantly outperforms all other approaches on a large collection of 500 binary decision problems varying in five levels of difficulty. The success of our algorithm demonstrates the potential of using meta-predictions to leverage latent expertise in environments where forecasters’ expertise cannot otherwise be easily identified.

## 1. Introduction

The fact that judgments can be improved by aggregating predictions across forecasters in a crowd has been well-known for over a century [1]. Simple averaging is a common approach to aggregating probabilistic forecasts and works well when forecasters have the same level of expertise. However, in practice, expertise is rarely constant across forecasters [2, 3]. A number of aggregation approaches have been developed to identify and leverage differences in expertise using forecasters’ past performance on questions with known outcomes [4, 5] and forecasters’ past contributions to the crowd forecast [6]. Unfortunately, information regarding past performance may often be unavailable because collecting forecasters’ responses to a set of relevant questions can be very time-consuming, costly, or otherwise impractical.

In a recent paper, Prelec, Seung, and McCoy [7] developed an innovative algorithm that uses meta-predictions—predictions about what others will predict—to correct for biases in the crowd where information regarding past performance is unknown. Their surprisingly popular (SP) algorithm predicts that the outcome that is more popular than the crowd expects (i.e., the surprisingly popular outcome) to be the correct answer.

In the current paper, we explore an alternative way of using meta-predictions to improve probabilistic forecasts. We propose the meta-probability weighting (MPW) algorithm, which weights the probabilistic forecasts of each forecaster by using the absolute difference between their prediction and their meta-prediction of the average prediction of others. As shown in our theoretical model discussed in the S1 Appendix, the weight assigned to each forecaster in the MPW algorithm is proportional to the absolute difference between the forecaster’s prior and the forecaster’s posterior in a Bayesian framework where forecasters receive private signals and share a common prior. Thus, forecasters with more informative private signals will be weighted more in the algorithm than those with less informative signals. Although this reweighting does not guarantee that the probabilistic forecast generated by the meta-probability weighting algorithm is closer to the truth than the simple average on a question-by-question basis, it does ensure that experts—individuals who have access to a more informative information system—will have higher expected weights than novice in crowds containing both types of individuals. Since experts will have better forecasts than novices on average, we hypothesize that the MPW algorithm will yield better probabilistic forecasts in the aggregate across many problems.

We test the performance of an extremised version of our algorithm against three current forecasting approaches in the literature—the extremised simple average, an extremised version of the minimal pivoting procedure of Palley and Soll [13], and the $p_{cs}^{\prime \prime }$ aggregator of Satopää, Pemantle, and Ungar [8]—using a large collection of 500 binary decision problems varying in five levels of difficulty. As discussed below, these alternative algorithms aim to improve the aggregate forecasts by correcting for the sharing or overlap in common information between forecasters. We find that the new algorithm outperforms all three alternative algorithms. We find that this outperformance is driven by improved performance on more difficult questions where there is likely to be heterogeneity in expertise.

The rest of this paper is organized as follows. In Section 2, we provide a formal definition of the MPW algorithm and discuss the theoretical properties of the algorithm. In Section 3, we describe our experimental design, the analyses we plan to conduct, and formally define each alternative aggregation approach. In Section 4, we examine the performance of each aggregation approach both generally and at the dataset level. Finally, in Section 5, we review the implications of these findings and the contribution it provides to the literature. The S1 Appendix contains our theoretical model while the S2 Appendix contains a comparison of the MPW algorithm and alternatives using the NCAA Men’s basketball dataset of Palley and Soll [13].

## 2. The MPW algorithm

Let *X* be the event space with events *X*_1_,*X*_2_,…*X*_*K*_ where *K* is the total number of events. Let *P*_*i*,*k*_ be the probability forecast of the *i*^th^ forecaster for the *k*^th^ event and let $M_{i,k}^{P}$ be this forecaster’s meta-prediction of the average forecast of others. Then, the probabilistic forecast made by the MPW algorithm, *T*_*MPW*_(*X*_*k*_), is given by

$$T_{MPW}(X_{k})=\sum_{i=1}^{N_{k}}W_{i,k}P_{i,k}$$ (1)

where *N*_*k*_ is the total number of forecasters for the *k*th event and

$$W_{i,k}=\frac{\left|P_{i,k}-M_{i,k}^{P}\right|}{\sum_{i=1}^{N_{k}}\left|P_{i,k}-M_{i,k}^{P}\right|}.$$ (2)

Note that by construction, the weights for each event *k* sum up to 1.

The weights for the MPW algorithm are informed by our theoretical model developed in the S1 Appendix. In our theoretical model, individuals share a common prior about the likelihood that the answer is true and receive private signals from one of two information systems that are ranked in terms of their informativeness. We allow the prior to be biased—as might be the case if forecasters receive a commonly observed public signal and update their beliefs to an informed common prior before receiving their private signals—but assume that signals are independent after conditioning on the state. We also assume all forecasters have common knowledge about the likelihood of a randomly selected forecaster receiving each potential signal in the true state and the false state. This assumption implies that two forecasters who receive the same private signal will have the same meta-prediction about the reports made by others.

We define an expert as an individual who receives a signal from the more informative information system and a novice as an individual who receives a signal from the less informative one. We show that under our theoretical assumptions, the weight of an individual is zero if the individual’s prior is equal to his or her posterior and that individual weights are increasing linearly in the distance between a forecaster’s prior and his or her posterior. In this sense, individuals with more informative private signals will be weighted more than individuals with less informative private signals. Since experts have a more informative signal than a novice on average, we can use Blackwell’s Theorem [21–25] to show that the expected weight of an expert is greater than the expected weight of a novice. We predict that the overweighting of experts in the algorithm will improve probabilistic forecasts in the aggregate.

## 3. The experiment

To test the MPW algorithm, we conducted an online experiment where we presented participants with US grade school true-or-false general science statements varying on five predefined levels of difficulty. We selected problems which varied systematically in difficulty because they provide a natural environment in which the level of expertise in the crowd varies accordingly. Our theoretical model predicts that the MPW algorithm is likely to offer the greatest improvement over simple averaging on moderate-difficulty and high-difficulty forecasting problems, where crowds are likely to contain forecasters with latent expertise. In contrast, the MPW algorithm is likely to provide little-to-no benefit over simple averaging on low-difficulty problems, where most forecasters are likely to be experts.

### 3.1 Experimental design

We generated 500 science statements at a US primary and secondary grade school level. Questions and content were adapted from worksheets on the Education Quizzes website (http://www.educationquizzes.com/us), and then converted into true or false statements. Approximately 2–3 questions were taken from each worksheet from the Biology, Chemistry, Geography, Physics, and General Science categories, spanning from grades 1 to 12, broken up into five levels of difficulty (grades 1 and 2; grades 3, 4, and 5; grades 6, 7, and 8; grades 9 and 10; and grades 11 and 12). We coded “difficulty 1” as the lowest difficulty level, and “difficulty 5” as the highest difficulty level. We treated each set of 100 questions of the same difficulty as an individual dataset. An example of a statement in difficulty 1 was “Omnivores only eat meat”. In contrast, difficulty 5 contained statements such as “Sound waves and electromagnetic waves are examples of longitudinal waves”. The full set of experiment questions, participant responses, and analysis code (for the MATLAB program, please see https://www.mathworks.com/products/matlab.html) are included in the supplementary information files.

The experiment was approved by the Melbourne School of Psychological Sciences Human Ethics Advisory Group (Ethics ID: 1647855.1) and all experiments were performed in accordance with the relevant guidelines and regulations. We recruited 500 respondents from Amazon Mechanical Turk; only respondents inside the US were able to participate in the experiment. Participants were paid a flat fee of USD $4.00 for completing the survey and all participants provided their written informed consent before completing the survey. The survey was conducted on the Qualtrics platform. Before beginning the experiment, participants were first required to answer three basic logic questions to deter any non-human agents from responding to the survey. Participants were then asked to answer each question as honestly as they could and without cheating (e.g., by looking up any of the questions online). Forty-one individuals who reported cheating at the task were excluded from the analyses; analyses were conducted on the data of the remaining 459 participants.

Participants completed 100 trials each, with each trial comprising one statement that was either true or false. Half the statements at each level of difficulty were true, and the other half were false. Participants were asked to provide their predictions about (a) whether the statement was more likely to be true or false, (b) what percentage of other forecasters would predict the statement to be true, (c) the probability that the statement was true, and (d) what the average probability estimated by other forecasters would be. Participants who provided votes that were inconsistent with their probability forecasts (i.e., voting “true” but predicting a probability <50% of the statement being true, or voting “false” but predicting a probability >50% of the statement being true) were excluded from the analysis from that particular question. Each participant saw 20 statements from each level of difficulty, and statements were presented in one of five randomized orders. Participants who took part in any of our previous experiments were excluded from participating.

### 3.2 Alternative algorithms and planned analyses

Our main algorithm of interest is the meta-probability weighting (MPW) algorithm, which weights forecasters’ probability forecasts by the normalized absolute difference between their probability forecasts and their meta-predictions about the average probability forecasted by others. Our comparison set also includes three other approaches from the literature: the simple average, the $p_{cs}^{\prime \prime }$ aggregator [8], and the minimal pivoting procedure [13]. The details of each aggregation approach used are shown in Table 1.

**Table 1:** Details of each aggregation approach used. The name, formula, and description for each probabilistic aggregation approach used in this paper. The notation for each aggregation approach is explained in the main text above, excluding the $p_{cs}^{\prime \prime }$ aggregator, for which, due to its complexity, we refer readers to the original paper by Satopää et al. [8].

Aggregation approach	Formula	Description
Simple average	$T_{\mu }(X_{k})=\sum_{i=1}^{N_{k}}\frac{P_{i,k}}{N_{k}}$	Simple unweighted average of all individual forecasts in the crowd.
$p_{cs}^{\prime \prime }$	$T_{p_{cs}^{\prime \prime }}(X_{k})=\Phi \left(\frac{\frac{1}{(N-1)\lambda +1}\sum_{i=1}^{N}X_{B_{i}}}{\sqrt{1-\frac{N\delta }{(N-1)\lambda +1}}}\right)$	Revealed Aggregator for the Gaussian Model under compound symmetry–see Satopää et al. [8] for details.
Minimal Pivoting	$T_{MP}(X_{k})=\sum_{i=1}^{N_{k}}\frac{P_{i,k}+({P_{i,k}-M}_{i,k}^{P})}{N_{k}}$	Simple average corrected by the minimal pivoting procedure [13].
Meta-probability Weighting (MPW)	$T_{MPW}(X_{k})=\sum_{i=1}^{N_{k}}\frac{|P_{i,k}-M_{i,k}^{P}|P_{i,k}}{\sum_{j=1}^{N_{k}}\left|P_{j,k}-M_{j,k}^{P}\right|}$	Weighted average of forecasters’ probability forecasts, where weights are calculated from the normalized absolute difference between their probability forecasts and their meta-predictions about the average probability forecasted by others.

The $p_{cs}^{\prime \prime }$ aggregator of Satopää, Pemantle, and Ungar [8] was designed to correct for the conservative bias that is consistently seen in probabilistic forecasting [9, 10, 11, 12]. As discussed in detail in [8], the algorithm is informed by a *partial information* framework that models the amount of information overlap in forecasters. While estimation of the parameters of the full model is possible with records of forecasters’ past performance, a simpler model—the $p_{cs}^{\prime \prime }$ aggregator—can be applied by assuming that the information available to forecasters is compound symmetric, such that forecasters’ information sets have the same size and the amount of pairwise overlap is constant. Assuming compound symmetry, the $p_{cs}^{\prime \prime }$ aggregator is able to estimate the amount of overlap in information between forecasters and therefore correct for this overlap by extremizing probability forecasts such that forecasts of low probabilities are shifted closer to 0 and forecasts of high probabilities are shifted closer to 1. Empirically, the authors found that the $p_{cs}^{\prime \prime }$ aggregator outperformed simple averaging and also both log-odds and probit aggregators on a large dataset of real-world geopolitical forecasting problems from the ACE forecasting tournament.

Palley and Soll [13] utilized a different approach, the *minimal pivoting* procedure, to correct for bias in the aggregated crowd forecast due to the sharing of information by adjusting the average forecast using forecasters’ meta-predictions about the average forecast of others. The authors showed that the optimal correction (or *pivot*) for this bias depends on the structure of shared information between forecasters. For example, the optimal amount of pivoting for a crowd of laypeople will differ to the optimal amount of pivoting for a crowd of experts. As the structure of shared information for a given problem may be unknown to the decision-maker beforehand, the authors proposed the use of a minimal pivoting procedure, which provides a conservative correction relative to the optimal pivoting procedure when the information structure is known. The authors tested the minimal pivoting procedure across four studies and found that minimal pivoting outperformed simple averaging on both a cost-estimation task and sports prediction problems.

While we could have applied these aggregation approaches directly, many previous studies have highlighted the consistent need for extremisation in the probabilistic forecasting domain [9, 10, 11, 14, 15, 16]. We therefore considered two versions of each algorithm: the standard version and a version augmented using the extremisation function used by Baron et al. [9] and others before them [10, 11]:

$$t(p)=\frac{p^{a}}{p^{a}+{\left(1-p\right)}^{a}}$$ (3)

where *p* is the original aggregated probability forecast, *t(p)* is the recalibrated probability, and *a* is the recalibration parameter, which determines the strength of the transformation. This function extremises probability forecasts when *a* > 1 and anti-extremises when 0 < *a* < 1. Baron et al. [9] conducted a large-scale study where over 2,000 people were asked to estimate the probabilities of outcomes to international events such as political elections occurring a future date. Baron et al. [9] found that the optimal parameter value for this function was approximately *a* = 2.5 in crowds containing expert forecasters, who, on average, were under-confident and therefore needed to be extremised to become optimally calibrated. For this reason, we selected this parameter value in advance and applied it to each aggregation approach. Extremisation improved forecasts for the simple average, MPW algorithm, and minimal pivoting procedure, but not for the $p_{cs}^{\prime \prime }$ aggregator, which already produced extremised forecasts [8]. In our results, we report the comparison between the extremised version of the MPW algorithm and both the standard and extremised versions of each other aggregation approach.

In line with Budescu and Chen [6] and Chen et al. [17], we compare the performance of the MPW algorithm and other probabilistic aggregation approaches using a transformed Brier score:

$$S_{i}=100-100\sum_{k=1}^{K}\frac{{\left(D(o_{k})-{T(X}_{i,k})\right)}^{2}}{K},$$ (4)

where *S*_*i*_ is the score of the *i*^th^ forecaster (or algorithm) averaged across *K* total events, *D*(*o*_*k*_) is the outcome variable for the *k*^th^ event (equals 1 if the event is true and 0 if false), and *T*(*X*_*i*,*k*_) is the probability assigned to that outcome being true by that forecaster (or algorithm). This scoring rule has a straightforward interpretation where scores range from 0 to 100, with 100 being a perfect forecast over all events. Importantly, this linear transformation of the Brier score retains the same functional form as the original and is strictly proper [18]. Strictly proper scoring rules are conventional measures of performance in probabilistic forecasting and are useful because they ensure that performance of the probability forecasts, measured as some sort of score, is optimized only by forecasts of the true probability. The use of scoring rules in assessing forecasts thus encourages forecasters to be careful and truthful in making their forecasts, in order to maximize their score.

We assess statistical significance between predictions of different aggregation approaches using 95% confidence intervals (CIs), which indicate, firstly, a significance difference when the null hypothesis value (*H*_*0*_ = 0) is not contained within the interval, and secondly, a plausible range for the size of the effect. We compute 95% confidence intervals for paired differences in transformed Brier score between the MPW algorithm and each other approach using the empirical bias-corrected and accelerated (BCa) bootstrap [19] using 10,000 bootstrap samples. Confidence intervals were computed using the standard *bootci* function in the MATLAB program. We have included the experimental data and MATLAB code for the analyses and plots from this paper in the supplementary information files.

## 4. Results

**Fig 1:**
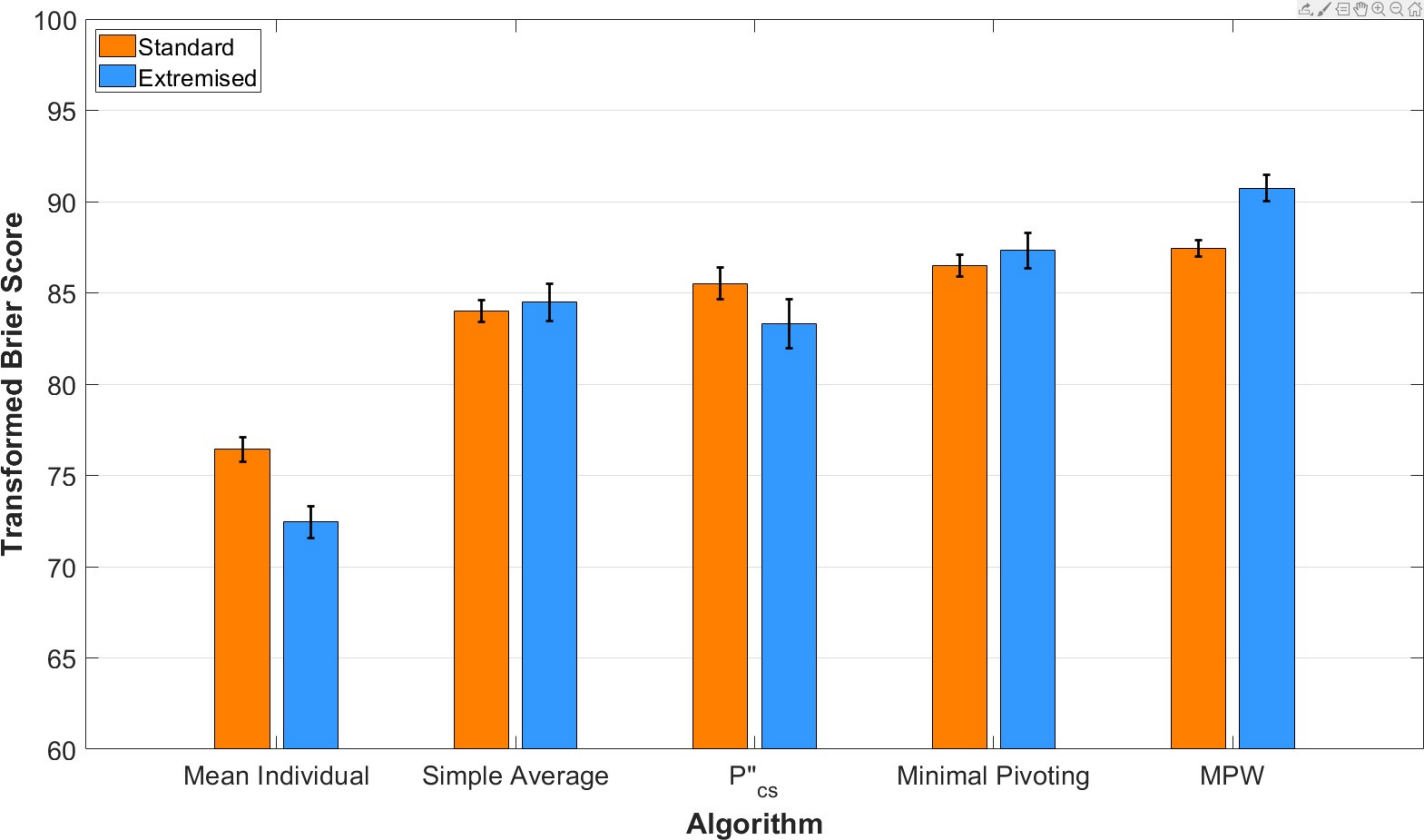
Overall performance of the standard vs. extremised versions of each aggregation approach. The mean transformed Brier score over a total of 500 US grade school problems spanning five levels of difficulty. Error bars indicate the standard error. The standard version of each approach generates probabilistic forecasts according to their formulae shown in Table 1. The extremised version of each approach transforms these predictions using a simple extremisation function **[9]**. The extremised MPW algorithm significantly outperforms both the standard and extremised versions of every other aggregation approach.

Fig 1 shows the mean performance for each aggregation approach across the 500 problems. After extremisation, the MPW algorithm generated significantly better predictions overall than: the standard mean individual by 14.22 points (bootstrap 95% CIs for paired mean difference in score: [13.04, 15.36]), the extremised mean individual by 18.20 points (95% CI: [16.86, 19.57]), the standard simple average by 6.64 points (95% CI: [5.61, 7.63]), the extremised simple average by 6.23 points (95% CI: [4.91, 7.62]), the standard $p_{cs}^{\prime \prime }$ aggregator by 5.04 points (95% CI: [3.83, 6.44]), the extremised $p_{cs}^{\prime \prime }$ aggregator by 7.24 points (95% CI: [5.40, 9.33]), the standard minimal pivoting procedure by 4.21 points (95% CI: [3.37, 4.98]), and the extremised minimal pivoting procedure by 3.43 points (95% CI: [2.47, 4.47]). The MPW algorithm was therefore highly effective at generating probabilistic forecasts across a range of low-difficulty to high-difficulty decision problems.

**Fig 2:**
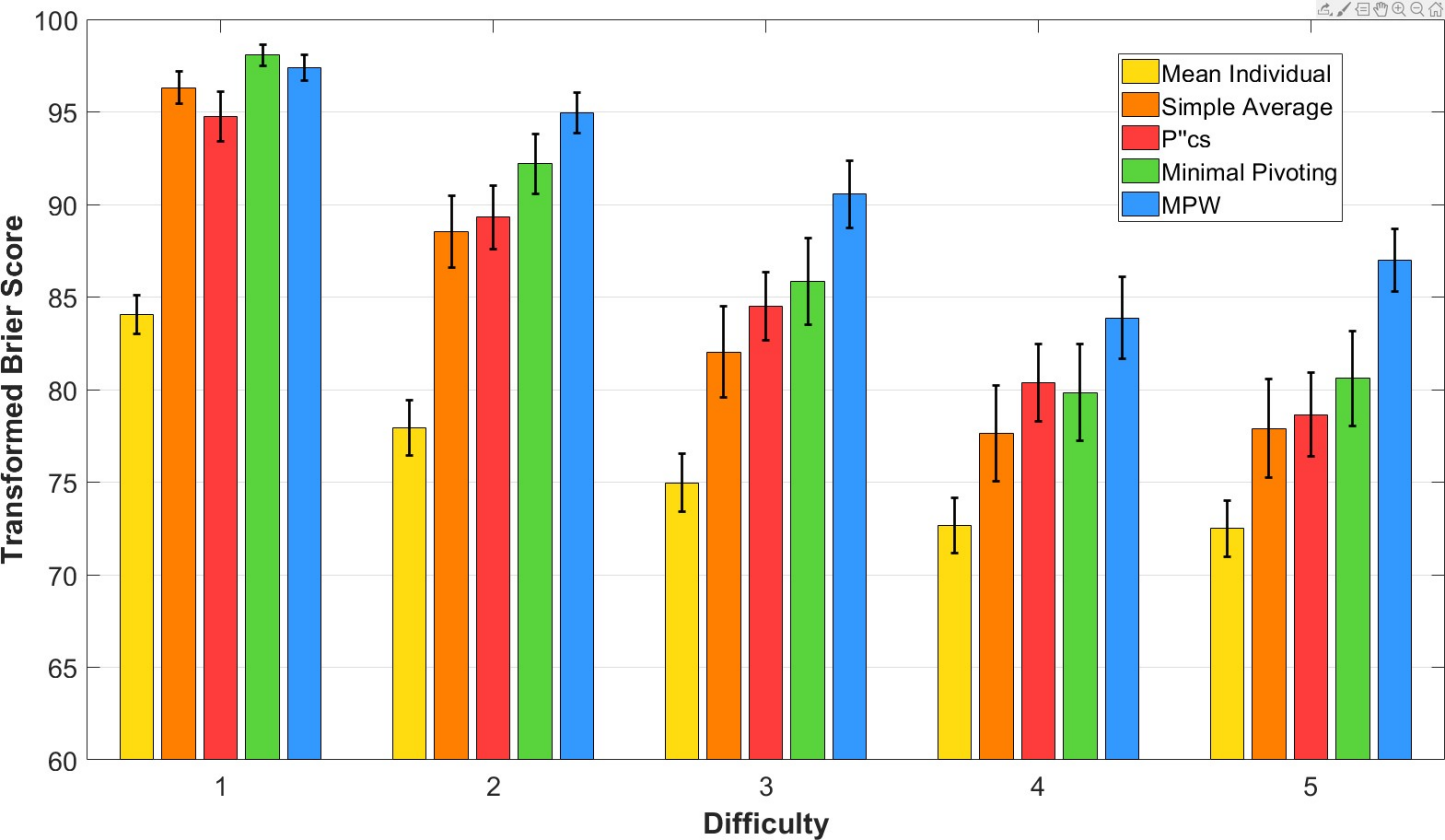
Performance of each aggregation approach on each level of difficulty. The mean transformed Brier score for each level of difficulty of US grade school problems. Error bars indicate the standard error. The extremised MPW algorithm (blue bar) outperforms the best-performing version of all other aggregation approaches on problems from difficulties 2 to 5. The 95% CIs for mean difference in score between the extremised MPW algorithm and each other aggregation approach is shown in Table 2.

We examined whether the improvement offered by the MPW algorithm over simple averaging varied across different problem difficulties. As the MPW algorithm leverages latent expertise, we would expect it to offer the greatest improvement over simple averaging on moderate-difficulty and high-difficulty forecasting problems, where the crowd is likely comprised of both experts and novices. Fig 2 shows the mean performance of the best-performing versions of each aggregation approach separately for each of the five difficulty levels. Table 2 shows the mean difference in transformed Brier score between the extremised MPW algorithm and each other approach for each difficulty. While the extremised MPW algorithm outperformed all other approaches on the problem sets from difficulties 2 to 5, this improvement was only significant for all comparisons from difficulty 2, 3, and 5.

**Table 2:** 95% Confidence intervals for the mean difference in the transformed Brier score between the extremised MPW algorithm and the standard and extremised versions of each other aggregation approach. Asterisks indicate where the difference in score was statistically significant at the *α* = .05 level according to the paired mean difference in transformed Brier score using the BCa bootstrap [19].

Aggregation approach	Version	Difficulty 1	Difficulty 2	Difficulty 3	Difficulty 4	Difficulty 5
Mean individual	Standard	[11.22, 15.58]*	[14.44, 19.65]*	[12.79, 18.19]*	[7.93, 13.89]*	[12.04, 16.79]*
	Extremised	[12.97, 18.48]*	[17.32, 23.78]*	[16.90, 23.08]*	[13.16, 19.23]*	[16.49, 22.04]*
Simple average	Standard	[3.62, 6.95]*	[6.25, 10.44]*	[5.63, 10.23]*	[1.06, 6.78]*	[5.24, 9.59]*
	Extremised	[-0.85, 3.03]	[3.54, 9.75]*	[5.86, 11.57]*	[3.23, 9.38]*	[5.79, 12.72]*
$p_{cs}^{\prime \prime }$	Standard	[0.47, 6.11]*	[3.02, 8.98]*	[3.66, 8.49]*	[0.53, 6.75]*	[5.42, 12.03]*
	Extremised	[-1.01, 6.63]	[2.32, 11.16]*	[5.07, 13.25]*	[3.73, 12.41]*	[7.69, 18.29]*
Minimal pivoting	Standard	[1.44, 3.94]*	[3.61, 6.80]*	[3.20, 6.89]*	[-0.17, 4.53]	[3.68, 7.22]*
	Extremised	[-2.23, 0.47]	[0.80, 5.11]*	[2.88, 6.88]*	[1.87, 6.50]*	[3.75, 9.47]*

* indicates where *p* < .05

The extremised MPW algorithm performed particularly well relative to other approaches on the problems in the highest difficulty level. For example, the extremised MPW algorithm outperformed simple averaging by approximately 9 points in score, which was approximately three times as large an improvement compared to that offered by the next best approach, the extremised minimal pivoting procedure. Consistent with our predictions, the extremised MPW algorithm also performed equally well as other aggregation approaches on the lowest difficulty level. Our empirical findings are thus highly consistent with the predictions of our theoretical model. These results provide strong evidence for the MPW algorithm’s mechanism to leverage latent crowd expertise, a mechanism that is most effective on moderate-difficulty to high-difficulty forecasting problems where the crowd is likely to be comprised of both experts and novices.

One explanation for our results is that the parameter values chosen for the extremisation function were simply better suited for the extremised MPW algorithm than these other aggregation approaches. Although we based our choice of parameter values from previous results from other authors [9], it could be the case that these values were simply optimized for the MPW algorithm and not the other aggregation approaches. To address this concern, we conducted additional post-hoc analyses to investigate whether optimally recalibrating these other aggregation approaches could allow them to outperform the extremised MPW algorithm. We optimally recalibrated each other aggregation approach using that approach’s responses to other forecasting problems (i.e., using cross-validation when past performance is known). For each approach, we used leave-one-out cross-validation to estimate the optimal parameter (*a*) in the recalibration function adapted from Baron et al. [9]. For each training set, we tested a range of values for a from 0 to 10 in increments of 0.01 and selected the value that maximized the score of that approach, which we then applied to the training event. We repeated this process separately for each of the 500 questions in the dataset, and for each of the five aggregation approaches. For statistical inference, we used the BCa bootstrap [19] with 10,000 bootstrap samples to compute 95% CIs for the mean paired difference in score between aggregation approaches.

**Fig 3:**
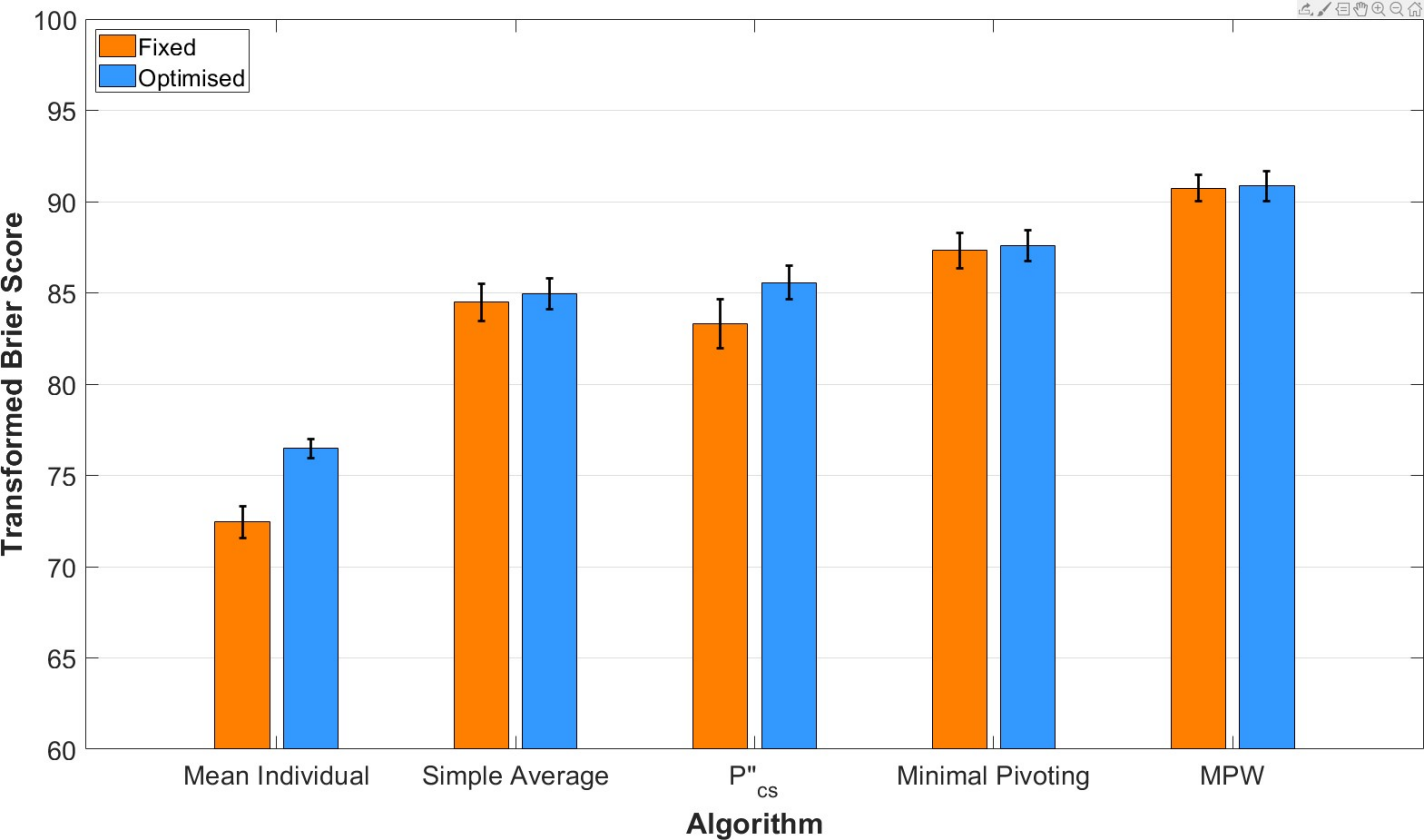
Performance of each aggregation approach using cross-validated recalibration parameters. This figure shows the mean performance of each approach using the fixed parameter value *a* = 2.5 (orange bars) vs. optimal recalibration parameters estimated via cross-validation (blue bars). Error bars show the standard error.

Fig 3 shows the performance of these other aggregation approaches once they have been optimally recalibrated. While optimizing the recalibration function for these other approaches improved their performance, the extremised MPW algorithm, which was not optimally recalibrated, still offered significantly better predictions than any other approach. Comparing the mean performance of the fixed version of the extremised MPW algorithm to the other optimally recalibrated approaches, we find that the extremised MPW algorithm outperforms each other approach even when they have been optimally recalibrated. The fixed extremised MPW algorithm scored higher than the optimally-recalibrated simple average by 5.79 points (95% CI: [4.66, 6.94]), the optimally-recalibrated $p_{cs}^{\prime \prime }$ aggregator by 5.19 points (95% CI: [3.91, 6.65]), and the optimally-recalibrated minimal pivoting procedure by 3.15 points (95% CI: [2.33, 4.01]).

In the S2 Appendix, we also conducted a post-hoc analysis where we compared the extremized version of each aggregation approach included in this paper to a dataset containing forecasts about NCAA men’s basketball games that was collected by Palley & Soll [13]. In this dataset we find no significant difference between the performance of the extremised MPW algorithm, the extremised minimal pivoting mechanism, the $p_{cs}^{\prime \prime }$ aggregator, or the extremised simple average. The dataset does not appear to have any experts in it, which may account for the similar prediction of all four methods.

## 5. Discussion

In the current paper, we have developed a novel algorithm for leveraging forecasters’ expertise using forecasters’ meta-predictions about what other forecasters would predict. The extremised MPW algorithm allows decision-makers to generate accurate probabilistic predictions even when the forecasters’ past performance is unavailable. The extremised MPW algorithm is also computationally simple, which may be appealing to decision-makers that are unfamiliar with more-sophisticated aggregation approaches that require structural estimation of latent parameters [20]. While previous research have demonstrated how meta-predictions can be used to correct for crowd biases [7], or used to identify the structure and extent of shared information in the crowd [13], no studies to date have shown that forecasters’ meta-predictions can be used to derive weights that quantify latent expertise. The extremised MPW algorithm is therefore theoretically distinct from existing approaches such as the $p_{cs}^{\prime \prime }$ aggregator [8] and the minimal pivoting procedure [13], which seek to improve forecasts by modelling and correcting for the overlap in information between forecasters.

The current paper provides a valuable contribution in demonstrating that this empirical quantity can be used to produce probabilistic forecasts that outperform existing aggregation approaches in the literature. In particular, the extremised MPW algorithm outperforms other existing aggregation approaches that can be applied on forecasting problems where the forecasters’ past performance is unknown: simple averaging, the $p_{cs}^{\prime \prime }$ aggregator [8], and the minimal pivoting procedure [13]. Relative to these other approaches, the extremised MPW algorithm performs particularly well for the more difficult forecasting problems, where leveraging latent expertise is likely to be most effective. Decision-makers who are faced with difficult forecasting problems may therefore find the extremised MPW algorithm an attractive alternative over existing aggregation approaches.

## Supporting information

S1 Appendix Theory appendix for understanding how the MPW algorithm leverages expertise [21, 22, 23, 24, 25]. (PDF)

S2 Appendix Testing the MPW algorithm on Palley & Soll (2018)’s NCAA Men’s basketball dataset. (PDF)

S1 File Experimental questions, participant responses, and analysis code. (ZIP)
