# Supplementary material for: Using meta-predictions to identify experts in the crowd when past performance is unknown
Source: PLoS One. 2020 Apr 24;15(4):e0232058. doi: 10.1371/journal.pone.0232058 (PMC7182234; doi:10.1371/journal.pone.0232058)
Supplement: S1 Appendix — (PDF) [file pone.0232058.s001.pdf]

Appendix S1. Theory appendix for understanding how the MPW algorithm leverages expertise

In this appendix, we investigate the conditions under which the meta-probability weighting (MPW) algorithm is able to leverage expertise by weighting the probabilistic predictions of experts more than novices. We show that under very general conditions, an individual with a more informative signal about the true state will be weighted more heavily by the algorithm than an individual with a less informative signal. This implies that from an ex-ante perspective, an individual who has access to a more informative information system will have a higher expected weight than an individual with a less informative information system.

1. Preliminaries

As is common in the information economics literature, we model expertise by considering an environment in which individuals have access to an information system (often called an experiment) in which they receive a signal that they use to update an initial prior belief [21–24]. Experts and novices are distinguished by the informativeness of their information system but are identical in all other dimensions.

We consider a Bayesian model in which a crowd of forecasters share a common prior $p(T)$ that an event is true. Each forecaster receives a private signal S, that is a random variable taking on real value realizations in the set $\{s_1, \ldots, s_m\} \cup \{s_\varnothing\}$ where $0 \leq s_1 < s_2 < \cdots < s_m \leq 1$ and $s_1 < s_\varnothing < s_m$. As our outcome space is binary, it is without loss of generality that we normalize the signals so that their value is equal to the posterior belief that an event is true. That is, $s_k := p(T|s_k)$. We let $s_\varnothing$ represent the case where an individual receives an uninformative signal so that $s_\varnothing := p(T)$.

The potential signals that an individual can receive is based on the information service that each forecaster has access to. We assume that there are two alternative information services — one for experts and one for the novices — with likelihood matrices $[Q^E_{oj}]_{2\times(m+1)}$ and $[Q^N_{oj}]_{2\times(m+1)}$. Each element of the first row of Q^E and Q^N represents the probability that the signal is s_j given the outcome is $o = T$. Likewise, each element of the second row of Q^E and Q^N represent the probability that the signal is s_j given the outcome is $o = F$. For ease, we will denote the first row elements with T and the second row elements with F. Thus $Q^E_{Tj} := Q^E_{1j} = p(s_j|T)$ while $Q^E_{Fj} := Q^E_{2j} = p(s_j|F)$.

We note two important features of an information service. First, an information service acts as a transition matrix from a state of nature to a signal and thus $\sum_j Q^\tau_{oj} = 1$ for each row $o \in \{T, F\}$ and information service $\tau \in \{N, E\}$. Second, upon receiving a message from information service τ, agents revise their priors using Bayes rule. For any signal that occurs with positive probability (i.e., where $Q^\tau_{Tj} + Q^\tau_{Fj} > 0$), the posterior belief that the event is true is given by

$$p(T|s_j) = \frac{p(T)Q^\tau_{Tj}}{p(T)Q^\tau_{Tj} + p(F)Q^\tau_{Fj}}.$$

By construction, this is equal to s_j for all signals that occur with positive probability.

We assume that the proportion of experts in the crowd is known to all parties and given by $\theta \in [0, 1]$. We also assume that the properties of Q^E and Q^N are common knowledge. We make two additional assumptions regarding the information services used by novices and experts:

Assumption 1 *Information service Q^E is more informative than information service Q^N: there exists a non-negative stochastic matrix $Z = [Z_{ki}]_{(m+1)\times(m+1)}$ such that*

$$Q^N = Q^E Z.$$

Assumption 1 says that when Q^E is more informative than Q^N, $Q^N_{oi} = \sum_k Q^E_{ok} Z_{ki}$. As we are multiplying across the rows of Q^E, we can interpret Z_{ki} as the conditional probability that when message k is received by Q^E, message i was received by Q^N. Thus $Z_{ki} = p(s_i|s_k)$ and Q^E is more informative than Q^N if it is possible to garble the signals of Q^E and generate Q^N. Note that Z is a non-negative stochastic matrix with $\sum_i Z_{ki} = 1$.

Assumption 2 *Experts and Novices draw independent signals: for a signal s_i from Q^N and a signal s_k from Q^E,*

$$p(s_i, s_k) = p(s_i|T)p(s_k|T)p(T) + p(s_i|F)p(s_k|F)p(F)$$

Assumption 2 implies that the information services are ranked but that signals from the two information services are independent once we condition for the state. Thus, for any signal s_i drawn from $Q^t, t \in \{N, E\}$, and any signal s_j drawn from $Q^\tau, \tau \in \{N, E\}$,

$$p(s_i|s_j) = \frac{p(s_i, s_j)}{p(s_j)} = \frac{p(s_i|T)p(s_j|T)p(T) + p(s_i|F)p(s_j|F)p(F)}{p(s_j)}.$$

Rearranging Bayes Rule, it is the case that:

$$\frac{p(T)p(s_j|T)}{p(s_j)} = p(T|s_j) = s_j$$

and thus

$$p(s_i|s_j) = p(s_i|T)s_j + p(s_i|F)(1 - s_j) = Q^t_{Ti}s_j + Q^t_{Fi}(1 - s_j). \tag{1}$$

We note that Assumption 2 is also sufficient for the monotone likelihood ratio property (MLRP) to hold for signals between any two information services. This property implies that when an individual receives a high signal, he believes that other forecasters are also more likely to receive a high signal.

2. The Expected Contribution of Experts and Novices

We now turn to the question of how the MPW algorithm weights experts and novices. A forecaster with signal s_k will make a probabilistic forecast of s_k. Thus, given an outcome state o,

the expected prediction from information service Q^t is given by

$$P(Q^t|o) = \sum_{\{i|s_i \geq 0\}} Q^t_{oi} s_i.$$

Aggregating over both information systems, the expected prediction of the population in state o is given by

$$P(\theta|o) = \sum_{\{i|s_i \geq 0\}} \theta P(Q^E|o) + (1-\theta) P(Q^N|o).$$

In the absence of any information service, the probabilistic forecast of each individual would be $s_\varnothing$. By the law of total expectations, the posteriors are a mean-preserving spread of the prior, and thus we have

$$s_\varnothing = s_\varnothing P(Q^\tau|T) + (1 - s_\varnothing) P(Q^\tau|F).$$

for $\tau \in \{E, N\}$. This also implies that

$$s_\varnothing = s_\varnothing P(\theta|T) + (1 - s_\varnothing) P(\theta|F)$$

and that

$$P(\theta|F) = \frac{s_\varnothing}{1 - s_\varnothing}[1 - P(\theta|T)]. \tag{2}$$

A forecaster with signal s_k's meta-prediction about the others is equal to

$$M(\theta|s_k) = s_k P(\theta|T) + (1 - s_k) P(\theta|F).$$

Substituting in for $P(\theta|F)$ using (2), the meta-prediction of an individual with signal s_k can be expressed as

$$M(\theta|s_k) = s_k P(\theta|T) + (1 - s_k) \frac{s_\varnothing}{1 - s_\varnothing}[1 - P(\theta|T)].$$

In the MPW algorithm, the weight of an individual is based on the difference between the individual's prediction and meta-prediction. For an individual with signal s_k,

$$s_k - M(\theta|s_k) = s_k - s_k P(\theta|T) - (1 - s_k) \frac{s_\varnothing}{1 - s_\varnothing}[1 - P(\theta|T)]$$

or, equivalently:

$$s_k - M(\theta|s_k) = \frac{s_k - s_\varnothing}{1 - s_\varnothing}[1 - P(\theta|T)]. \tag{3}$$

Note first that the difference between an individual's signal and his or her meta-prediction is zero at $s_\varnothing$ and is linearly increasing in s_k. This feature implies that the weight of an individual with signal s_k, proportional to $|s_k - M(\theta|s_k)|$, is directly related to the informativeness of the posterior that an individual holds relative to the prior. Thus, individuals with more informative posteriors (an ex-post notion of expertise) will be weighted proportionally more than individuals with less informative posteriors.

Aggregating over all possible signals that an individual might receive, the expected weight of an individual in information service Q^t is proportional to:

$$\mathbb{E}[w|Q^t] = \sum_{\{k|s_k>0\}} |s_k - M(\theta|s_k)|[s_{\varnothing}Q^t_{Tk} + (1 - s_{\varnothing})Q^t_{Fk}].$$

By Assumption 1, the information system of the expert is a mean-preserving spread of the signals of the novices. Noting that individuals with posteriors farther away from their prior will have a larger weight, it is possible to use Blackwell's theorem to show the following:

Proposition 1 *The expected weight of an expert is greater than the expected weight of a novice.*

We note that Proposition 1 does not rely on us having only two types of agents in the population and can be readily extended to an arbitrary number of information systems that are ranked by informativeness. Thus, the results here are general and are likely to hold in a wide variety of problems. We also note that, the MPW algorithm weights experts more than novices if we use an ex-ante notion of expertise based on informativeness of information systems or an ex-post notion of expertise based on the difference between an individual's posterior and their prior.

3. Proof of Proposition 1

Proof of Proposition 1: We begin this proof of Proposition 1 by stating Blackwell's Theorem [25]:

Blackwell's Theorem *For information service Q^E to be more informative than Q^N it is necessary and sufficient that the value of information in service Q^E is greater than the value of information in service Q^N for all sets of terminal actions, all utility functions, and all prior beliefs.*

By Assumption 1, Q^E is more informative than Q^N. Let the action set $a \in \{T, F\}$ correspond to voting on whether an answer is true or false, and consider a utility function $U(a, o)$ that maps actions and states of the world into outcomes. Let $U(T,T) = \frac{1}{1-s_\varnothing}[1-P(\theta|T)] - \frac{s_\varnothing}{1-s_\varnothing}[1-P(\theta|T)]$, $U(F,F) = \frac{1}{1-s_\varnothing}[1-P(\theta|t)] - [1-P(\theta|T)]$, $U(T,F) = -\frac{s_\varnothing}{1-s_\varnothing}[1-P(\theta|T)]$, and $U(F,T) = -[1-P(\theta|T)]$. Given a signal s_i, expected utility is maximized by choosing $a = T$ when $s_i \geq s_\varnothing$ and $a = F$ otherwise. The expected utility of this strategy when the posterior is less than $s_\varnothing$ is given by:

$$\begin{aligned}
\mathbb{E}[U(Q^t|s_i < s_\varnothing)] &= (1-s_i)U(F,F) + s_i U(F,T) \\
&= \frac{1-s_i}{1-s_\varnothing}[1-P(\theta|T)] - (1-s_i)[1-P(\theta|T)] - s_i[1-P(\theta|T)] \\
&= \frac{1-s_i}{1-s_\varnothing}[1-P(\theta|T)] - [1-P(\theta|T)] \\
&= \frac{s_\varnothing - s_i}{1-s_\varnothing}[1-P(\theta|T)].
\end{aligned}$$

Likewise, the expected utility of this strategy given a posterior greater than $s_\varnothing$ is

$$\begin{aligned}
\mathbb{E}[U(Q^t|s_i \geq s_\varnothing)] &= s_i U(T,T) + (1-s_i)U(T,F) \\
&= \frac{s_i}{1-s_\varnothing}[1-P(\theta|T)] - s_i\frac{s_\varnothing}{1-s_\varnothing}[1-P(\theta|T)] - (1-s_i)\frac{s_\varnothing}{1-s_\varnothing}[1-P(\theta|T)] \\
&= \frac{s_i}{1-s_\varnothing}[1-P(\theta|T)] - \frac{s_\varnothing}{1-s_\varnothing}[1-P(\theta|T)] \\
&= \frac{s_i - s_\varnothing}{1-s_\varnothing}[1-P(\theta|T)].
\end{aligned}$$

Thus, we can express the expected utility of an individual with signal s_i as

$$\mathbb{E}[U(Q^t|s_i)] = \frac{|s_i - s_\varnothing|}{1-s_\varnothing}[1-P(\theta|T)]. \tag{4}$$

By Blackwell's theorem, the expected utility of information service Q^E is higher than the expected value of information service Q^N for any utility function and any prior belief. Using an initial prior of $P(T) = s_\varnothing$, this implies,

$$\mathbb{E}[U(Q^E)] \geq \mathbb{E}[U(Q^N)]$$

By the law of iterated expectations:

$$\begin{aligned}\mathbb{E}[U(Q^t)] &= \sum_{\{i|s_i \geq 0\}} \mathbb{E}[U(Q^t|s_i)]p(s_i) \\ &= \sum_{\{i|s_i \geq 0\}} \mathbb{E}[U(Q^t|s_i)][s_\varnothing Q^t_{Ti} + (1-s_\varnothing)Q^t_{Fi}].\end{aligned}$$

Using the result in (4) above, this implies that

$$\mathbb{E}[U(Q^t)] = \sum_{\{i|s_i \geq 0\}} \frac{|s_i - s_\varnothing|}{1-s_\varnothing}[1 - P(\theta|T)][s_\varnothing Q^t_{Ti} + (1-s_\varnothing)Q^t_{Fi}]$$

Noting that this equation is identical to the expected weight of an individual from information sytem Q^E

$$E[w|Q^t] = \sum_{\{i|s_i \geq 0\}} \frac{|s_i - s_\varnothing|}{1-s_\varnothing}[1 - P(\theta|T)][s_\varnothing Q^t_{Ti} + (1-s_\varnothing)Q^t_{Fi}],$$

the result that $\mathbb{E}[U(Q^E)] \geq \mathbb{E}[U(Q^N)]$ immediately implies that $E[w|Q^E] \geq E[w|Q^N]$. ■
