# Supplementary material for: Using meta-predictions to identify experts in the crowd when past performance is unknown
Source: PLoS One. 2020 Apr 24;15(4):e0232058. doi: 10.1371/journal.pone.0232058 (PMC7182234; doi:10.1371/journal.pone.0232058)
Supplement: S2 Appendix — (PDF) [file pone.0232058.s002.pdf]

S2 Appendix. Testing the MPW algorithm on Palley & Soll (2018)'s NCAA Men's Basketball dataset.

In the main text, we have concentrated our analyses only on data that we have collected expressly for the purposes of testing the MPW algorithm. Out of interest, we also sought to test the MPW algorithm on datasets in the literature that have been collected by other authors. In this Appendix, we examine the performance of the extremised MPW algorithm on the data from Study 4 of Palley & Soll [1]. In their study, the authors elicited participants' forecasts for the probability that each team would win across 120 different games in the early rounds of the 2014, 2015, and 2016 NCAA Division I Men's Basketball Tournament. Participants were recruited via Amazon Mechanical Turk and completed the experiment via an online web survey.

The responses in their dataset in which we are interested are the (1) the team that the participant predicts would win a particular match, (2) the participants forecast for the probability that their selected team would win, and (3) their estimate of the average probability forecasted by other participants that the participant's selected team would win. These responses correspond to the responses that we had collected in our experiment in the main text.

We computed the predictions for each of the aggregation approaches in the main paper using the responses in this dataset. We calculated the transformed Brier score for each of the standard and extremised version of each aggregation approach from the main and plotted the mean results below. For statistical inference, we computed 95% CIs for the mean difference in transformed Brier score between each aggregation approach's forecasts using the BCa bootstrap 95% [2].

Fig S1 shows the performance of the standard and extremized version of each aggregation approach. The extremised MPW algorithm significantly outperformed the standard mean individual (95% CI for mean difference in transformed Brier score: [1.61, 6.49]), and the extremised mean individual (95% CI: [4.97, 8.70]). However, there was no significant difference in score between the extremised MPW algorithm and any other aggregation approach: the standard simple average (95% CI: [-2.72, 2.17]), the extremised simple average (95% CI: [-2.99, -0.07]), the standard p''_{cs} aggregator (95% CI: [-3.43, 0.86]), the extremised p''_{cs} aggregator (95% CI: [-2.33, 2.90]), the standard minimal pivoting procedure (95% CI: [-2.63, 1.60]), and the extremised minimal pivoting procedure (95% CI: [-2.23, 0.31]).

Although the extremised MPW algorithm was very effective for the datasets in the main text, it does not appear to improve forecasts in the NCAA forecasting domain. We suspect that this is due to a different distribution of experts and novices in the two datasets. In our US Grades dataset, there is a large dispersion in Brier scores across forecasters and clear experts and novices. As seen in Fig S2 below, which shows the distribution of mean Brier scores in the sample population, *ex post* performance is approximately normally distributed in the basketball dataset and there is no evidence for a distinct group of experts. The MPW algorithm therefore offered no significant advantage over simple averaging as there were few-to-no latent experts in the crowd for the algorithm to identify.

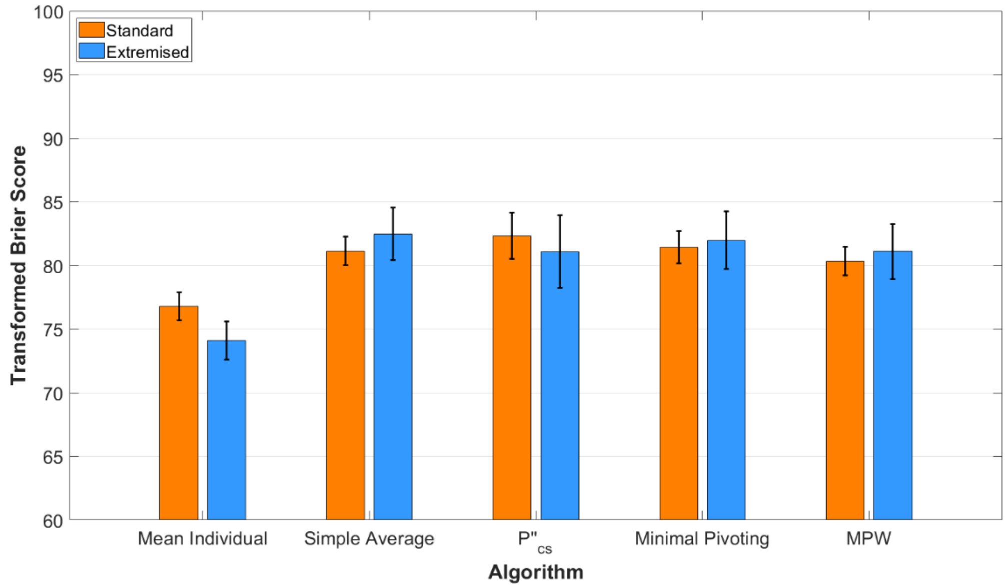

Fig S1. Overall performance of the standard vs. extremised versions of each aggregation approach in the Palley & Soll (2018) dataset. The mean transformed Brier score over a total of 120 events. Error bars indicate the standard error. The standard version of each approach generates probabilistic forecasts according to their formulae shown in Table 1. The extremised version of each approach transforms these predictions using a simple extremisation function [3]. There is no significant difference between the extremised MPW algorithm and any version of the other aggregation approaches.

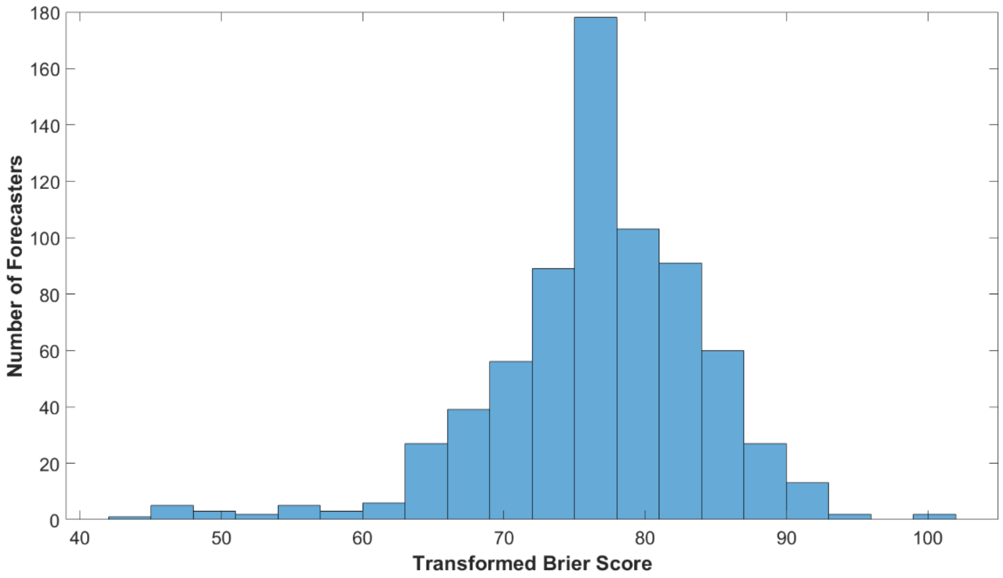

Fig S2. Distribution of forecasters' mean scores. Forecasters appear to be largely homogeneous in their *ex post* performance. There is no evidence for a distinct group of experts in the crowd.

References

[1] Palley AB, Soll JB. Extracting the Wisdom of Crowds When Information Is Shared. Management Science. 2019;65(5):2291–2309.

[2] Efron B. Better bootstrap confidence intervals. Journal of the American statistical Association. 1987;82(397):171–185.

[3] Baron J, Mellers BA, Tetlock PE, Stone E, Ungar LH. Two reasons to make aggregated probability forecasts more extreme. Decision Analysis. 2014;11(2):133–145.
