# Supplementary material for: Using meta-predictions to identify experts in the crowd when past performance is unknown
Source: PLoS One. 2020 Apr 24;15(4):e0232058. doi: 10.1371/journal.pone.0232058 (PMC7182234; doi:10.1371/journal.pone.0232058)
Supplement: S1 File — (ZIP) [file pone.0232058.s003.zip › AnalysisCode/readme.pdf]

Summary (‘AnalysisCode’ folder)

AnalysisCode.m contains the full analysis code containing all the results reported in *Probabilistic forecasting using crowd meta-predictions when past performance is unknown* (Martinie, Wilkening, and Howe, 2020). Running AnalysisCode.m runs all the functions in the analysis and generates all the outputs; this should take approximately 10-15 mins to run.

Dataset details (‘Raws’ subfolder)

Our results were collected over 5 iterations. Each XLSX file (r2a_rawdata.xlsx, r2b_rawdata.xlsx, r2c_rawdata.xlsx, r2d_rawdata.xlsx, r2e_rawdata.xlsx) contains a set of forecasters’ responses to 20 questions from five different difficulties from 1 (easiest) to 5 (hardest).

1. r2a_rawdata.xlsx
2. r2b_rawdata.xlsx
3. r2c_rawdata.xlsx
4. r2d_rawdata.xlsx
5. r2e_rawdata.xlsx

Each forecaster’s responses are contained in one row in the XLSX file. Each set of four columns in the XLSX files correspond to all forecasters’ responses to one question. The four columns correspond to the four responses we elicited for each question: (a) whether the statement presented was more likely to be true or false, (b) what percentage of other forecasters would predict the statement to be true, (c) the probability that the statement was true, and (d) what the average probability estimated by other forecasters would be. Thus, forecasters’ responses to the first question are in columns 1-4, the second question in columns 4-8, and so on.

Two additional files contain the ID for each question (1-500), the correct outcome for that question, the difficulty of that question, and the statement text for that question:

6. r2_outcomes.xlsx
7. QuestionsList.xlsx

Analysis Functions (‘Functions’ subfolder)

1. preprocessData.m – Generates a pre-processed ‘grdata.mat’ file from the raw data files.
2. GenPreds.m – Generates each model’s predictions for each event.
3. CVExtremisation.m – Uses leave-one-out cross-validation to estimate the optimal parameters separately for each event and for each model. Saves a ‘CVExtremisation.mat’ file (in the ‘Files’ subfolder) that contains the predictions from each optimally recalibrated model for each event.

Plotting functions (‘Functions’ subfolder)

1. plotMeanResultsFull.m – plots Figure 1 in the main text.
2. plotDatasets.m – plots Figure 2 in the main text.
3. plotExtermisation.m – plots Figure 3 in the main text.

Auxiliary functions ('Functions' subfolder)

1. BSCORE.m – computes the transformed Brier score for any probability forecast (1st input) based on outcome for that event (2nd input).
2. UNSCORE.m – computes the probability forecast from the transformed Brier score.
3. efunction.m – recalibrates probability forecasts (1st input) by the 'a' parameter (2nd input) – see Baron et al. (9).
4. colours.m – generates sets of RGB values for fabulous-looking plots.
5. fullfig.m – maximises the size of the figure window.

Data files ('Files' subfolder)

1. grdata.mat – MATLAB data file containing the forecasters' probability forecasts ('forecasts' variable), binary forecasts ('bf' variable), the outcome for each event ('outcomes' variable), meta-predictions about the average votes of others ('po' variable), meta-predictions about the average probability forecast of others ('pp' variable).
2. GenPreds.mat – MATLAB data file contain the predictions and scores for each version of each model ('preds' and 'results' variables) and the outcomes for each event ('outcomes' variable)'.
3. CVExtremisation.mat – MATLAB data file containing the predictions for each model using the fixed parameterisation (a = 2.5) in the recalibration function ('preds' variable) vs. optimised recalibration parameters for each model, on each event, estimated using leave-one-out cross-validation ('tpreds' variable).

R code ('R' subfolder)

1. analyseData.r – Generates probabilistic forecasts using the PCS aggregator - see Palley & Soll (13).
2. 1.csv – data file containing probability forecasts from every forecaster in the dataset and across all 500 events.
3. 1_preds.xlsx – the PCS aggregator's predictions for each of the 500 events.

Output ('Figures' subfolder)

1. Fig1.tif – Figure 1 in the main text.
2. Fig1_CIs.csv – 95% CIs and mean differences for each comparison in Figure 1.
3. Fig2.tif – Figure 2 in the main text.
4. Fig2&Table2_CIs.csv – 95% CIs and mean differences for the comparisons in Figure 2. Corresponds to Table 2 in the main text.
5. Fig3.tif – Figure 3 in the main text.
6. Fig3_CIs.csv – 95% CIs and mean differences for each comparison in Figure 3.
