# Supplementary figures and images for: Using meta-predictions to identify experts in the crowd when past performance is unknown
Source: PLoS One. 2020 Apr 24;15(4):e0232058. doi: 10.1371/journal.pone.0232058 (PMC7182234; doi:10.1371/journal.pone.0232058)

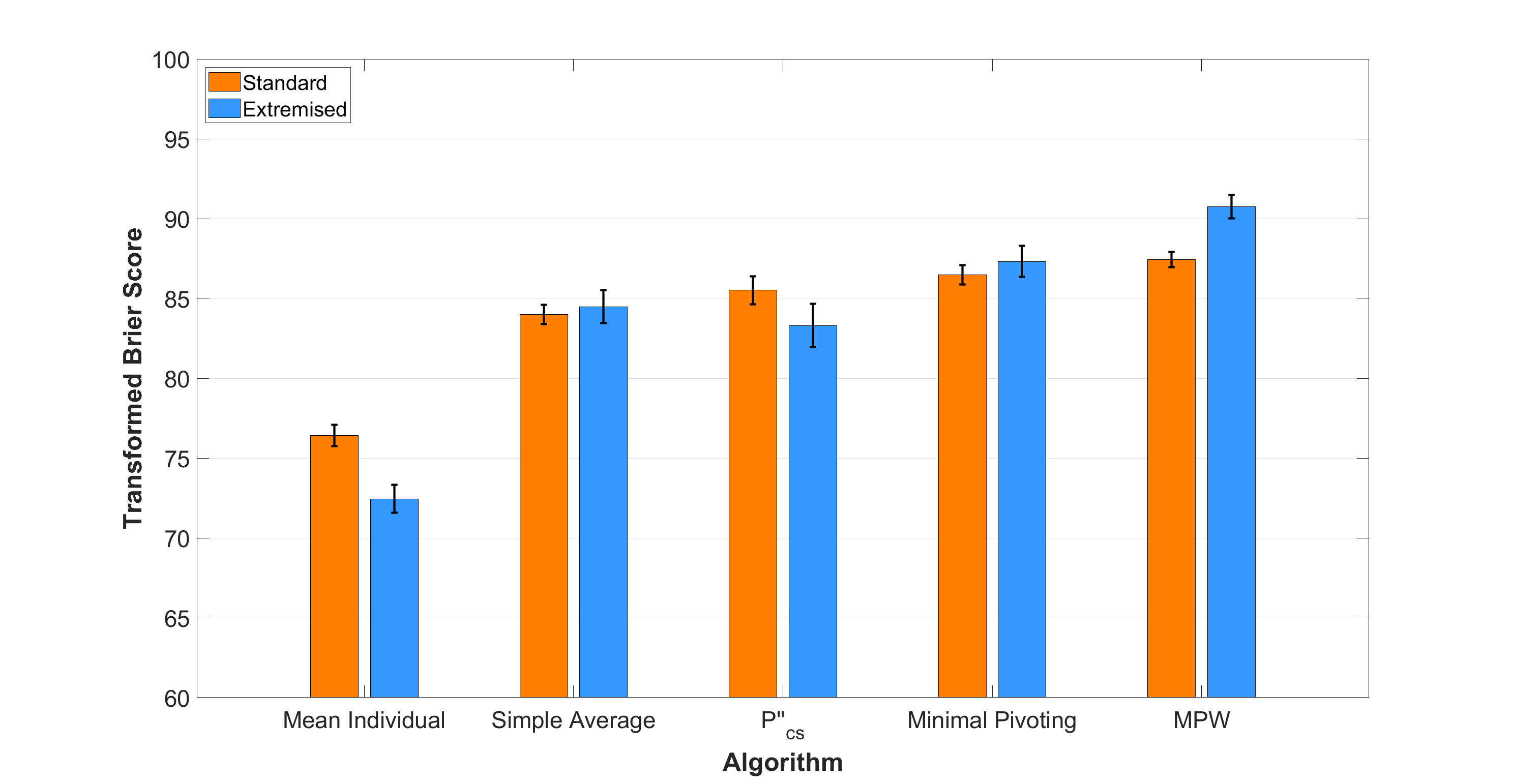

Supplement: S1 File — (ZIP) [file pone.0232058.s003.zip › AnalysisCode/Figures/Fig1.png]

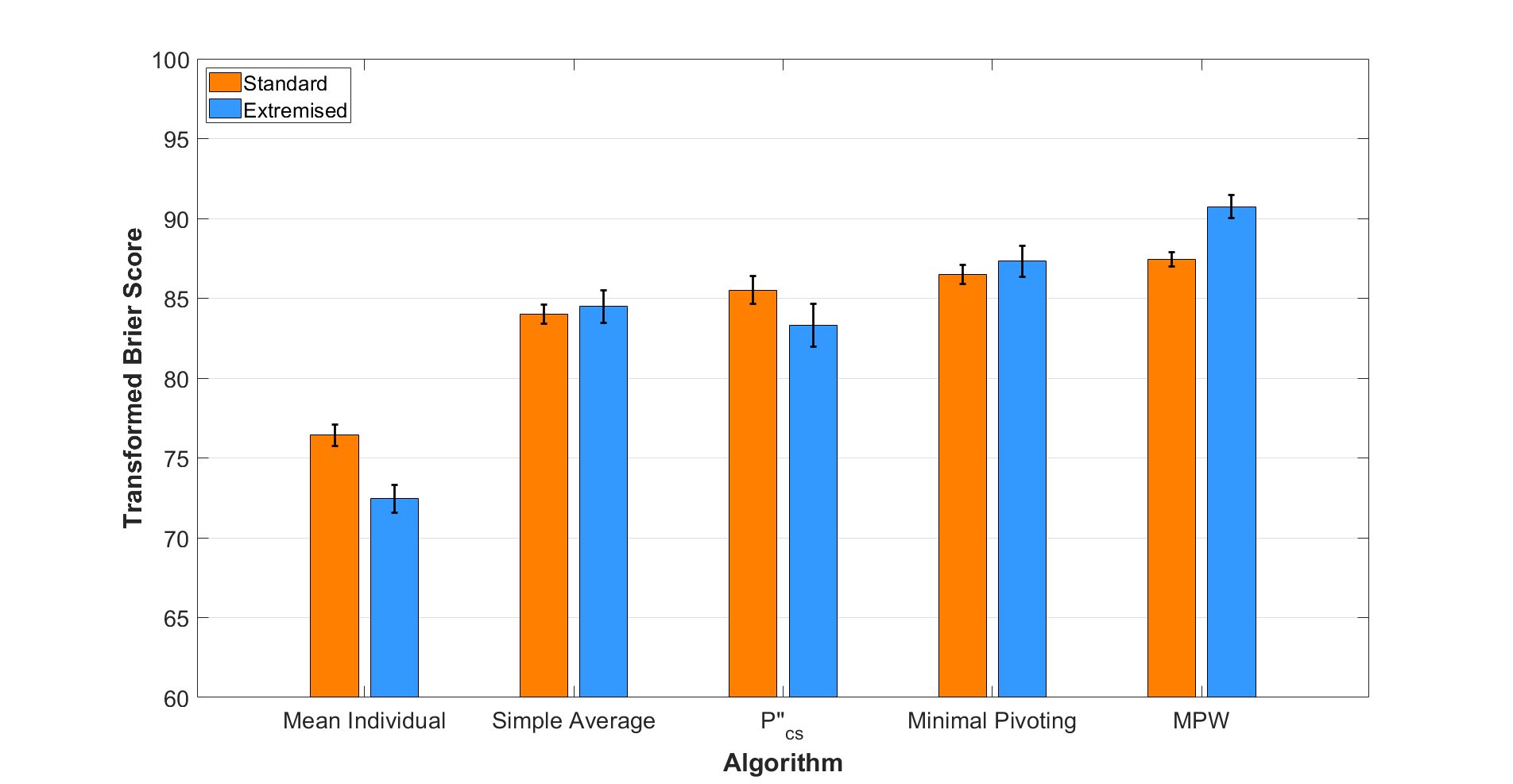

Supplement: S1 File — (ZIP) [file pone.0232058.s003.zip › AnalysisCode/Figures/Fig1.tif]

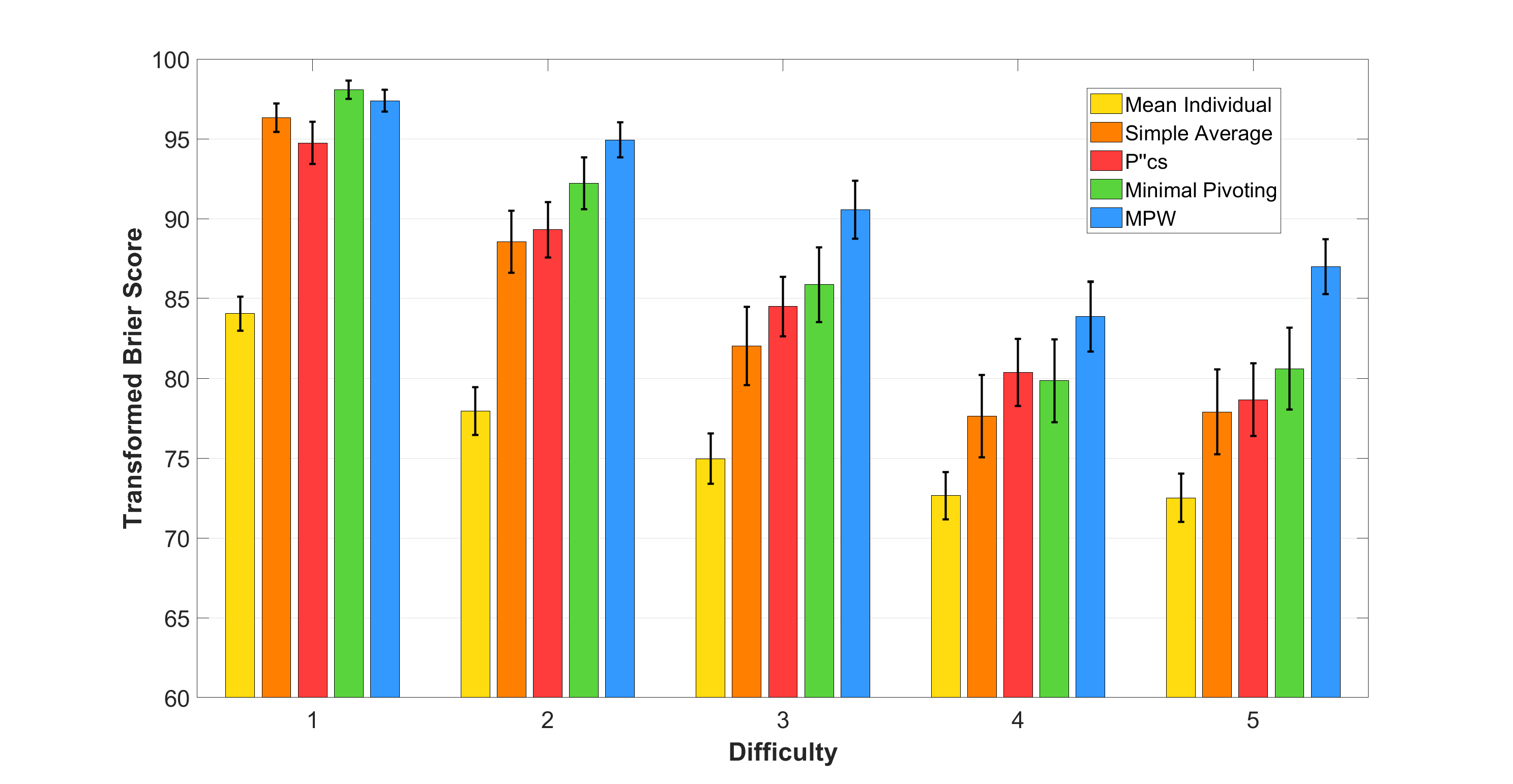

Supplement: S1 File — (ZIP) [file pone.0232058.s003.zip › AnalysisCode/Figures/Fig2.png]

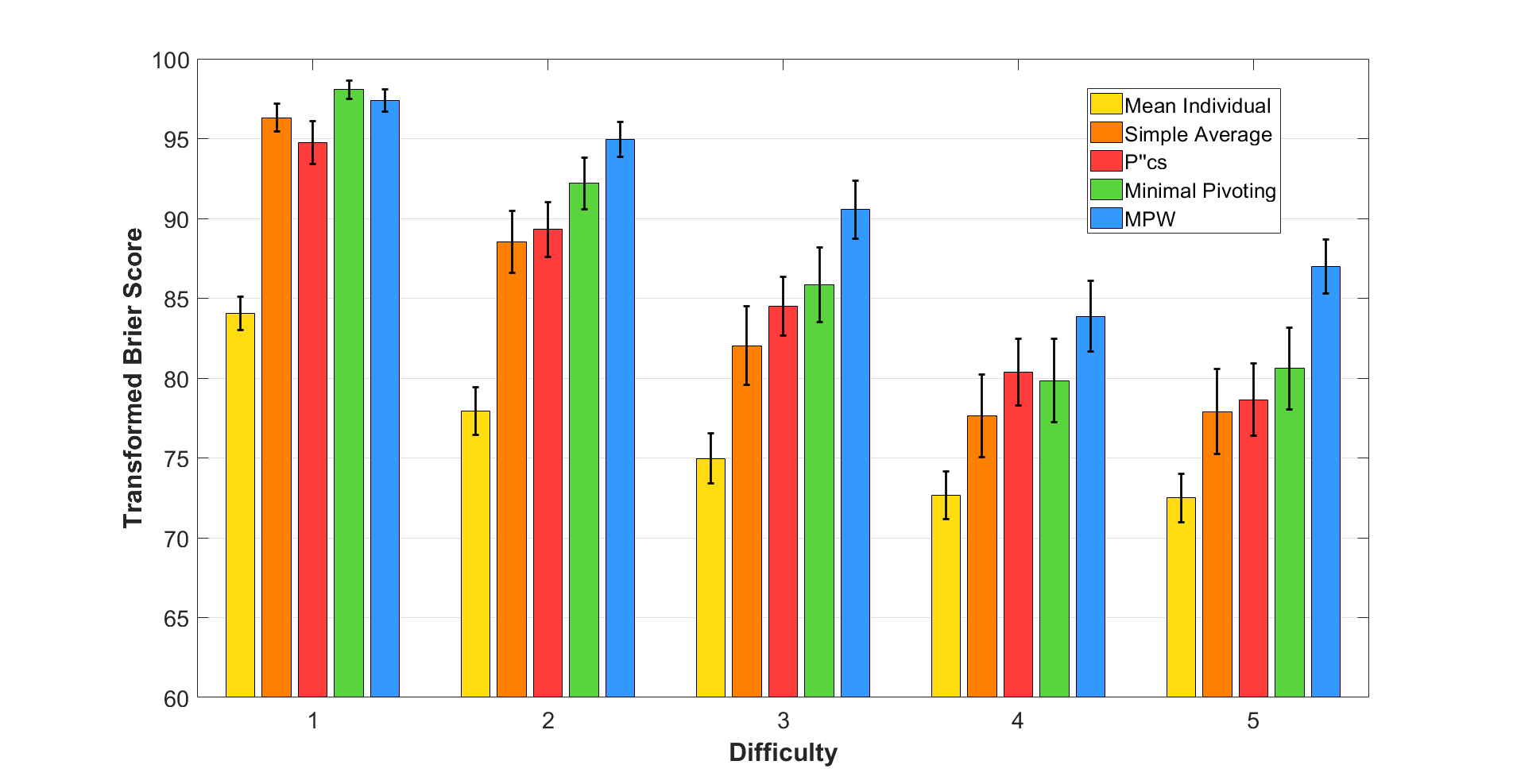

Supplement: S1 File — (ZIP) [file pone.0232058.s003.zip › AnalysisCode/Figures/Fig2.tif]

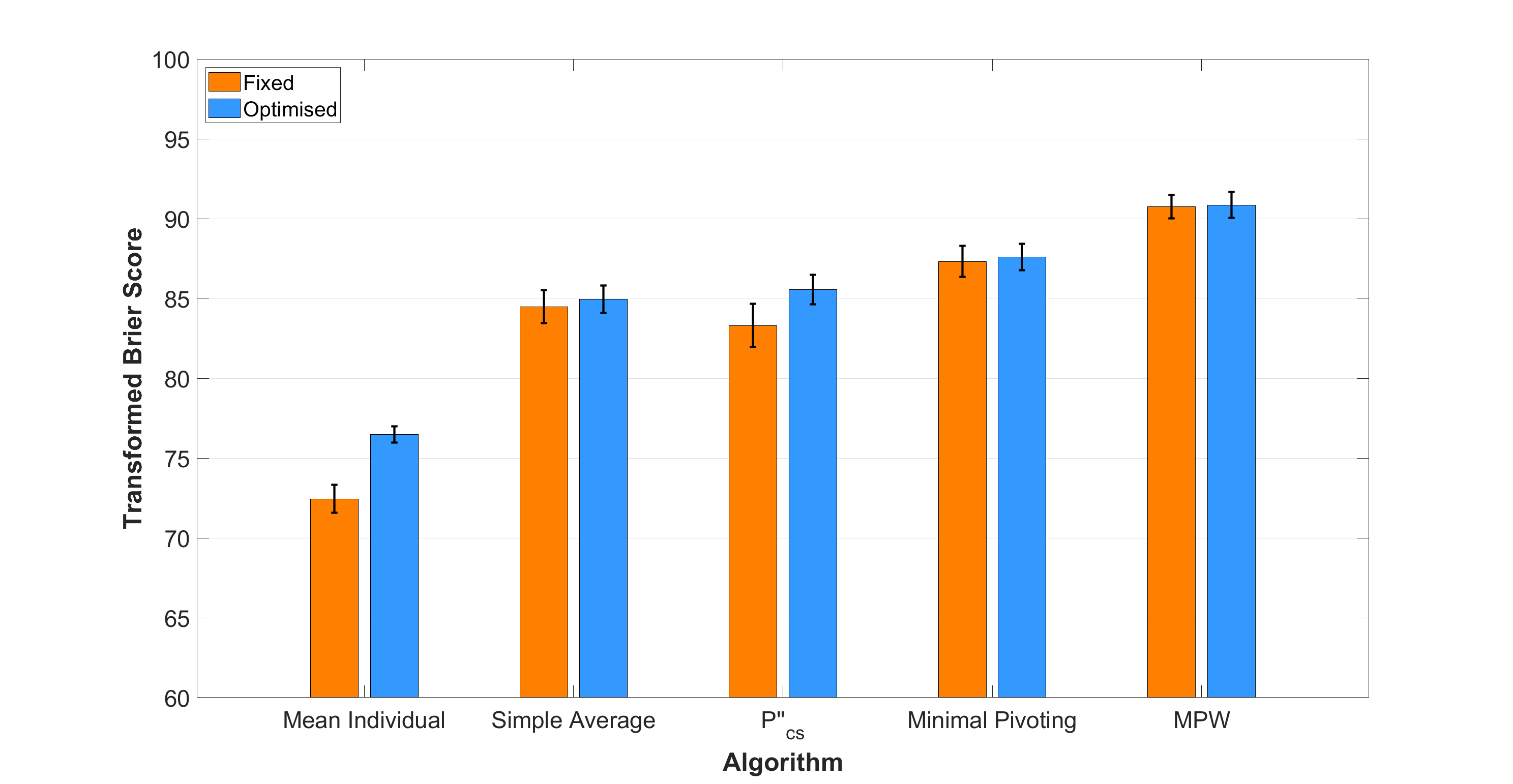

Supplement: S1 File — (ZIP) [file pone.0232058.s003.zip › AnalysisCode/Figures/Fig3.png]

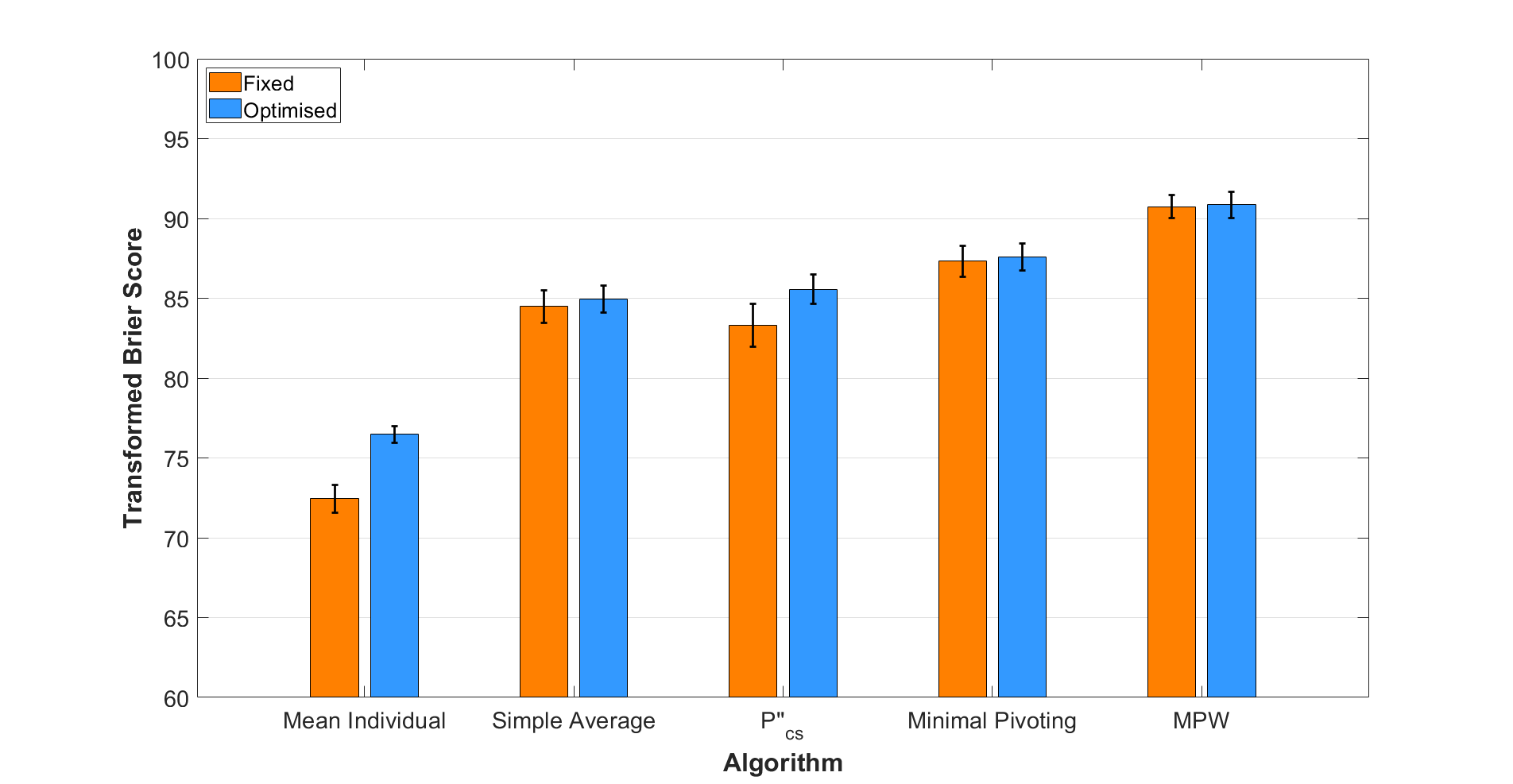

Supplement: S1 File — (ZIP) [file pone.0232058.s003.zip › AnalysisCode/Figures/Fig3.tif]
